# Effect of Oxidative Stress Induced by *Brevibacterium* sp. BS01 on a HAB Causing Species-*Alexandrium tamarense*

**DOI:** 10.1371/journal.pone.0063018

**Published:** 2013-05-08

**Authors:** Huajun Zhang, Xinli An, Yanyan Zhou, Bangzhou Zhang, Su Zhang, Dong Li, Zhangran Chen, Yi Li, Shijie Bai, Jinglin Lv, Wei Zheng, Yun Tian, Tianling Zheng

**Affiliations:** 1 State Key Laboratory of Marine Environmental Science and Key Laboratory of the Ministry of Education for Coastal and Wetland Ecosystems, School of Life Sciences, Xiamen University, Xiamen, China; 2 College of Chemical Engineering, Huaqiao University, Xiamen, China; Louisiana State University and A & M College, United States of America

## Abstract

Harmful algal blooms occur all over the world, destroying aquatic ecosystems and threatening other organisms. The culture supernatant of the marine algicidal actinomycete BS01 was able to lysis dinoﬂagellate *Alexandrium tamarense* ATGD98-006. Physiological and biochemical responses to oxidative stress in *A. tamarense* were investigated to elucidate the mechanism involved in BS01 inhibition of algal growth. Transmission electron microscope analysis revealed that there were some chloroplast abnormalities in response to BS01 supernatant. The decrease in cellular-soluble protein content suggested that cell growth was greatly inhibited at high concentration of BS01 supernatant. The increase in the levels of reactive oxygen species (ROS) and malondialdehyde contents following exposure to BS01 supernatant indicated that algal cells suffered from oxidative damage. The content of pigment was significantly decreased after 12 h treatment, which indicated that the accumulation of ROS destroyed pigment synthesis. Moreover, the decrease of Fv/Fm ratio suggested that in the photosynthetic system, the dominant sites producing ROS were destroyed by the supernatant of the BS01 culture. The activities of the antioxidant enzymes including superoxide dismutase and peroxidase increased in a short time and decreased slightly with increasing exposure time. A real-time PCR assay showed changes in the transcript abundances of two photosynthetic genes, *psb*A and *psb*D. The results showed that BS01 supernatant reduced the expression of the *psb*A gene after 2 h exposure, but the expression of the *psb*D gene was increased at concentrations of 1.0 and 1.5%. Our results demonstrated that the expression of the *psb*A gene was inhibited by the BS01 supernatant, which might block the electron transport chain, significantly enhancing ROS level and excess activity of the antioxidant system. The accumulation of ROS destoryed pigment synthesis and membrane integrity, and inhibited or ultimately killed the algal cells.

## Introduction

In recent decades, environmental pollution has become more and more serious. The continuous increase in the nutrients run-offs, especially nitrogen and phosphorus, to marine waters with eutrophication as consequence, is a worldwide phenomenon [1]. Harmful algal blooms (HABs), as one of the results of eutrophication, break the balance of the marine ecosystem and cause severe damage to the marine environment, threatening other organisms and even human health [2], [3]. *Alexandrium tamarense*, a notorious bloom-forming dinoflagellate which is associated with the largest number of paralytic shellfish poison (PSP) poisoning cases [4], [5], occurs in many coastal waters [6], and many physiological and ecological studies have been conducted to clarify the mechanism controlling blooms of this species [7], [8].

Aimed at terminating HABs, several approaches including physical and chemical methods have been carried out [9]–[11]. However, none of these approaches could resolve the problems completely, and they have the negative effects on high costs and secondary pollution [12]. Therefore, research on both economical and feasible approaches to algal removal has important theoretical and practical significance [13], [14]. Biological control agents such as bacteria [15]–[18], actinobacteria [19] and viruses [20], [21] have gained wide attention. In recent years, marine bacteria have been considered to be one of the key biological agents in the dramatic termination of phytoplankton blooms in coastal seawaters [6], [22], [23]. The actinomycetes are the main group of producers of marine bioactive substance, and because of their excellent application as antibiotics in medical science, actinomycetes are one of the most potential and effective biological agents to eliminate harmful algae. Algicidal actinomycetes that have been isolated at present are mostly *Streptomyces* species [24], [25].

Most reported algicidal bacteria exert algicidal activity by excreting extracellular substances, that is allelopathy. Allelopathy is the release of organic compounds by plants or bacterial species that affect other plants or bacterial species, which is regarded as a form of interference competition [26]. Current studies indicate that the mechanisms of allelochemical inhibition on algal growth take mainly four pathways: destruction of cell structure, influence on algal photosynthesis, or respiration, and alteration of enzymatic activities [27]–[29]. These allelochemicals exert toxic effects in aquatic organisms through oxidative stress, producing morphological alterations, degradation of DNA or protein and oxidation of membrane fatty acid that can lead to cell death. As an adaptative response, aquatic organisms increase antioxidant defenses to eliminate reactive oxygen species (ROS) and avoid oxidative damage. Superoxide dismutase (SOD), catalase (CAT), and peroxidases (PODs) and low molecular weight compounds, such as carotenoids and glutathione, are all included in antioxidant defenses [29], [30]. FischerellinA (FS), produced by *Fischerella muscicola*, can strongly inhibit the growth of cyanobacteria and other photosynthetic organisms, and conclude that FS acts at several sites: (1) effect on the rate constant of QA^-^ reoxidation; (2) primary photochemistry trapping; (3) inactivation of the PSII reaction center; and (4) segregation of individual units from grouped units, because the loss of the observed sigmoidicity corresponds to a decrease of cooperativity or grouping could stimulate the formation of separate pack units out of grouped units [31]. Previous studies note the alteration of gene expression of algae under the stress of allelochemicals. Shao *et al*. [32] have reported the gene expressions of *Microcystis aeruginosa* for *pr*x, *mcy*B, *psb*A, *rec*A, *grp*E, *fab*Z under pyrogallol stress. However, seldom do reports indicate the change of gene expression in *A. tamarense*.

Our previous research focused on the isolation and characterization of algicidal microbes and algicidal substances, and demonstrates that *Brevibacterium* sp. BS01 shows strong algicidal activity against *A. tamarense* [33]. In order to illustrate the mechanism of this actinomycete, further studies were carried out: (1) to monitor any morphological alteration of algal cells under transmission electron microscopy (TEM); (2) to investigate the effect on bio-molecules such as fatty acids and proteins; (3) to determine the activities of antioxidant enzymes as a response to oxidative stress; and (4) to explore the response of photosynthetic systems under the stress of BS01. The objective of our study was to elucidate the role of ROS, which might play its role as a signal to trigger cellular death under the algicidal actinomycete when it came to optimal concentration in the algal cells.

## Materials and Methods

### Actinobacteria Cultures and Supernatant Preparation

*Brevibacterium* sp. BS01 (Genebank No. GQ274005) was isolated from Pearl Bay (part of Xiamen Bay) in China [33]. Cells of BS01 were inoculated into Zobell 2216E broth (peptone 5 g/L, yeast extract 1 g/L, ferric phosphorous acid 0.1 g/L, dissolved in natural seawater, pH 7.6–7.8) followed by incubation for 48 h at 28°C. Then the cells were removed by centrifugation at 10,000×g for 10 min and the supernatant was filtered through a 0.22 µm Millipore membrane. The supernatant was collected and stored at −80°C.

### Algal Cultures and BS01 Supernatant Treatment

Cultures of the experimental alga, *A. tamarense* ATGD98-006, were supplied by the Algal Culture Collection, Institute of Hydrobiology, Jinan University, Guangzhou, China. The cultures were incubated in sterile f/2 medium (without silicate) prepared with natural seawater [34] at 20±1°C under a 12 h : 12 h light-dark cycle with a light intensity of 50 µmol photons m^−2^s^−1^. Exponential phase axenic cultures were used for further experiments.

Flasks (250 mL) were prepared and each of them contained 100 mL of sterile f/2 algal culture medium. BS01 supernatant as described above was added into axenic exponentially growing algal cultures at a ratio of 0.5% (v/v), 1.0% (v/v) and 1.5% (v/v) in triplicate and co-cultures in order to measure algicidal rate according to the reported formula [35], [36]. Autoclaved Zobell 2216E broth served as the control.

### Sample Preparation and Transmission Electron Microscopy

Algal cells were treated with BS01 supernatant for 8 h, and were then fixed for TEM. Samples were fixed overnight at 4°C in 0.1 M PBS buffer containing 2.5% glutaraldehyde (v/v) and post-fixed in 1% OsO_4_ in the same buffer for 2 h. The samples were then dehydrated through a graded ethanol series [30, 50, 70, 90 and 100% (v/v in ddH_2_O); 15 min at each concentration] followed by a graded ethanol:acetone series (3∶1, 1∶1, 1∶3, 0∶1; 15 min at each concentration) at 4°C and embedded in araldite resin. Sections (60–80 nm), obtained with an ultramicrotome, were stained in 3% acetic acid uranium-citric acid and viewed using a JEM2100HC (Japan) transmission electron microscope.

### Determination of ROS Levels

Intracellular ROS was detected using a fluorescent probe, 2′,7′- dichlorofluorescin diacetate (DCFH-DA), according to the report mehtod [37], but with slight modifications. 0.5 mL DCFH-DA (the final concentration in the mixture was 10 µM) was added to the cell particles and the mixture was incubated in an incubator at 37°C in the dark for 1 h and resuspended every 5 min during this time. Then the cells were washed three times with sterile f/2 medium immediately and finally suspended with 1 mL sterile f/2 medium. The fluorescence intensity was monitored using a spectrofluorometer with excitation wavelength at 485 nm and emission wavelength at 525 nm.

### Lipid Peroxidation and Antioxidative Enzyme Assays of of *A. tamarense*

Algal cells were pelleted using centrifugation at 3,000×g for 5 min followed by rinsing in 1 mL of PBS (50 mM, pH 7.8). After the pellets were resuspended in PBS, cell disruption was conducted using Ultrasonic Cell Disruption System (NingBo Scientiz Biotechnological Co., Ltd, China) (80 W, 5 s: 5 s, 40 times) below 4°C. Debris was removed using centrifugation at 10,000×g for 10 min at 4°C. 1 mL was used to assay cellular protein which was detected using the “Coomassie brilliant blue protein analysis kit” (Nanjing Jiancheng Bioengineering Institute, China), using bovine serum albumin as the standard. The rest was stored at −80°C until it was used to analyze the alteration of MDA (a byproduct of lipid peroxidation), and the activities of SOD and POD. All the analysis methods followed the kit’s Operation Manual from Nanjing Jiancheng Bioengineering Institute, China [38].

### Pigments and Chlorophyll Fluorescence Measurement

5 mL of algal culture was used to analyze the content of chloroplast pigments based on the report method [39], but with some modifications. The algal cells were collected, and washed with PBS (50 mM, pH7.8), and then the pigments were extracted using 95% ethanol at 4°C in the dark overnight followed by centrifugation at 4°C for 10 min at 8,000×g. The supernatant was used to measure absorbance values at wavelengths of 665 nm, 645 nm and 470 nm. The pigments were calculated according to the formulae [39]:
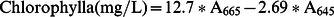


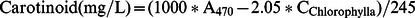
A_665_, A_645_ and A_470_ represent absorbance values at wavelengths of 665 nm, 645 nm, and 470 nm. C _Chlorophyll a_ represents the content of chlorophyll a (Chl a).

The PAM fluorescence measurements were performed using a PAM-CONTROL Fluorometer (Walz, Effeltrich, Germany). The cells were dark-adapted for 15 min before measurements under an actinic light of 3000 µmol photons m^−2^s^−1^ [40]. Fv/Fm is biomass independent and can be used as an indicator for the general level of fitness of photosynthetic organisms.

### RNA Extraction and Quantitative Real-time PCR Analysis

80 mL of 2 h treated algal culture was centrifuged at 3,500 rpm for 5 min at 4°C, and the cell pellets frozen at −80°C until RNA extraction. Total RNA was extracted using the RNAiso kit (TaKaRa Company, Dalian, China) following the manufacturer’s instructions. For reverse transcription, 1 µg of total RNA was mixed with Oligo (dT) primers and reverse transcriptase following the instructions of the reverse transcriptase kit (Fermentas, EU). Real-time PCR was carried out using an “SYBR® Premix EX Taq™ II” (TaKaRa Company, Dalian,China). The primer pairs for *psb*A, *psb*D are listed in Table 1. The following PCR program was used with two steps: one denaturation step at 95°C for 20 s and 45 cycles of 95°C for 10 s, followed by 60°C for 20 s and 72°C for 20 s. 18S rRNA was used as a reference gene to normalize the expression changes. The relative gene expression among the treatment groups was quantified using the 2^−ΔΔCt^ method [41].

**Table 1:** Primer pairs in *A. tamarense* for real-time PCR.

Gene	Sequence (5′-3′)
**18s**	F: 5′-TGGTGGAGTGATTTGTCTGGTT-3′
	R: 5′-CCTGTTATTGCCTCAAACTTCCTT-3′
***psb*** **A**	F:5′-CTACGATGTGAAATGGATGC-3′
	R:5′-CTTTTTCGGCACCTCTT-3′
***psb*** **D**	F:5′-ACAAACCTCCAAAAAGAACCCAACG-3′
	R:5′-GCAAGATTATCAACACCAGCAAACAG-3′

### Statistics

All data were presented as means ± standard error of the mean and were evaluated using one-way analysis of variance followed by the least significant difference test, with *p*<0.01 and *p*<0.05 (Origin 8.5 for Windows).

## Results

### Effect of BS01 Supernatant on Subcellular Structure

**Figure 1:**
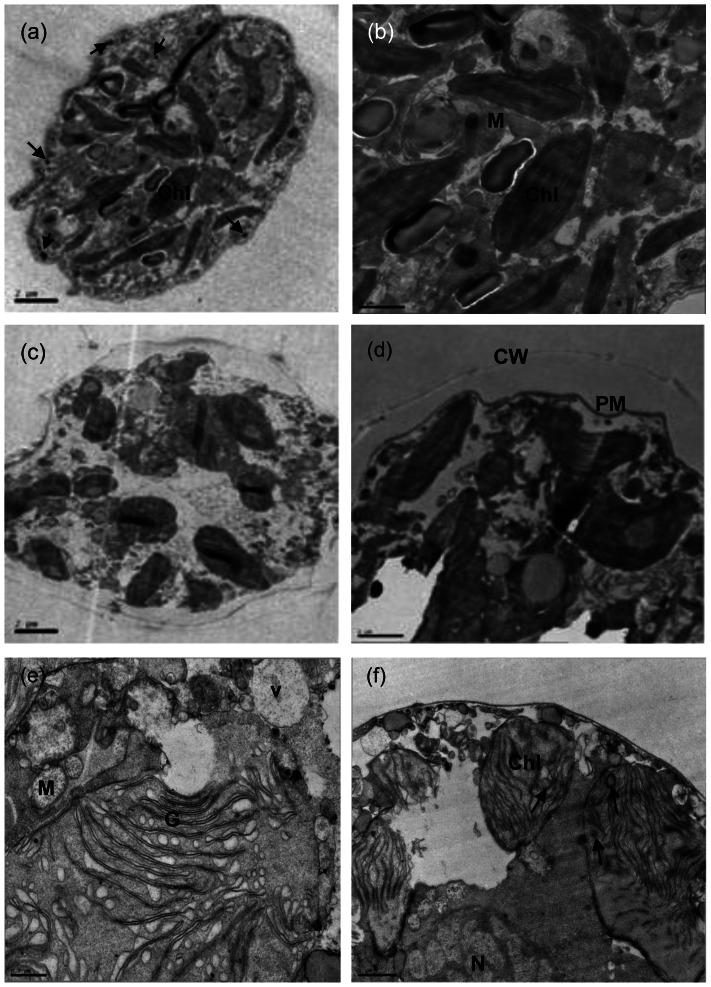
Ultrastructure of *A.*tamarense after exposure to BS01 supernatant for 8 h with the concentration of 1.0%. (a) overview of control cell, electron dense particles with arrows; (b) detail view of control cell; (c)–(f) details in the treated cell. Abbreviations: CW, cell wall; Chl, chloroplast; G: golgi body; M: mitochondrion; V: vacuole; N: nucleus; PM, plasma membrane; starch grains with arrows in (f). Bars (a), (c) 2 µm; (b), (d) and (f) 1 µm; (e) 0.5 µm.

TEM analysis revealed alterations of the ultrastructure of *A. tamarense,* with some cells losing the integrity of their organelles (Fig. 1c–f). Compared with the normal cells without exposure to 1.0% of BS01 supernatant for 8 h (Fig. 1a–b), these treated cells showed many differences in morphological characteristics and even structural damage (Fig. 1c–f). In control cells, multi-lobed intact chloroplasts in the cells and the cytoplasm were dense, and there were also fewer and smaller vacuoles (Fig. 1a), and moreover, electron dense particles were observed in the control cell. A detailed view of the chloroplast structure with starch grains and mitochondria with cristae was given in Fig. 1b. *A. tamarense* cells treated for 8 h with 1.0% BS01 supernatant showed extreme plasmolysis and vacuolization, and the structure and morphology of the cell wall was seriously affected in these cells (Fig. 1c–d). The number of multivesicular bodies was significantly increased (Fig. 1e), but the mitochondria and vacuoles were not affected in these cells. In contrast, the chloroplasts were severely damaged in treated cells with internal degradation (Fig. 1f). Disorganization and deformation of the chloroplast up to a complete disintegration of the thylakoids were observed. We also saw increased starch grains and rupture of the chloroplast in this cell. As exposure concentration increased (such as in the 1.5% BS01 supernatant), we observed more severe ultrastructural changes and even cell lysis under TEM (data not shown).

### Assays for Protein and ROS

**Figure 2:**
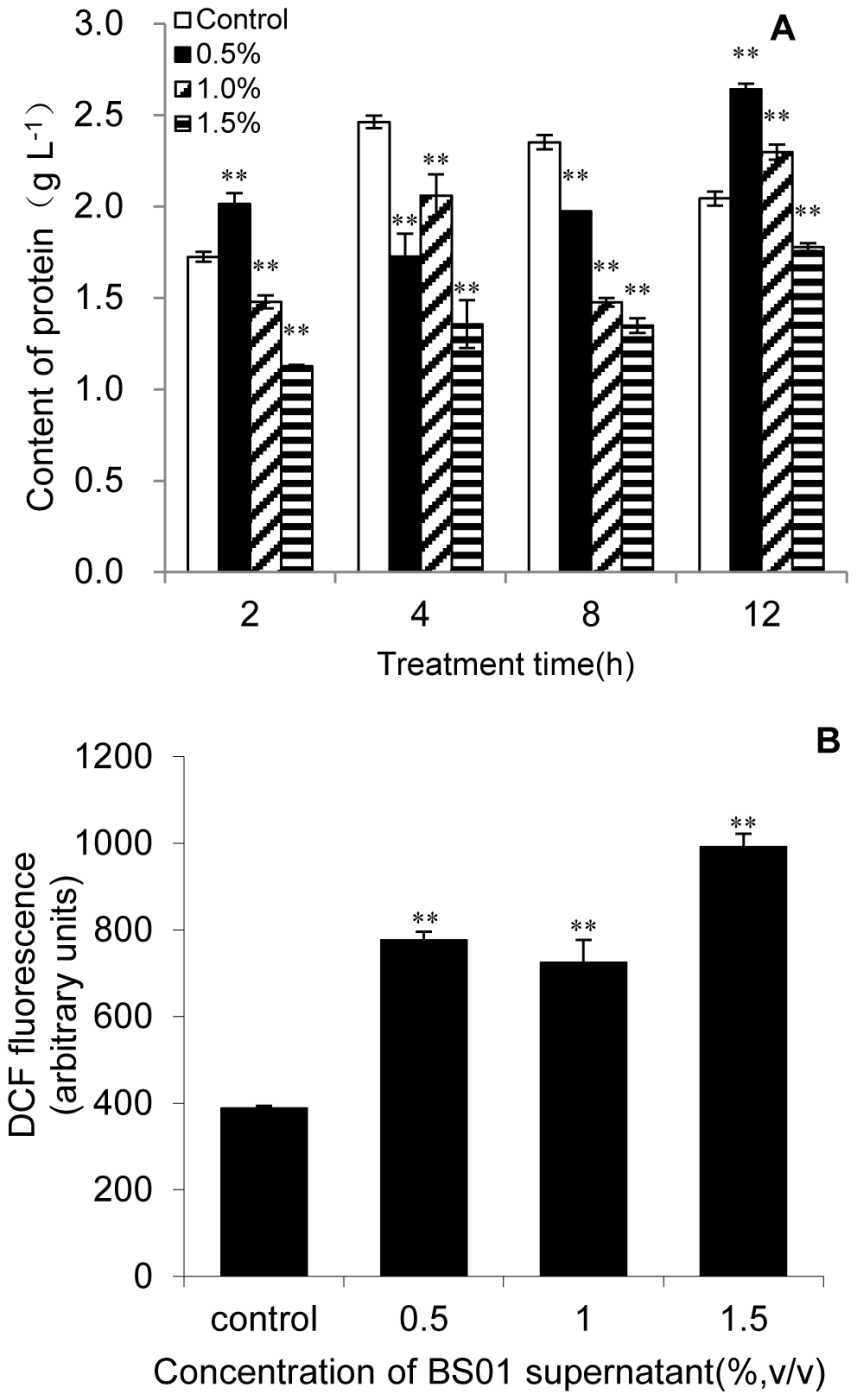
Effects of BS01 supernatant on cellular protein and ROS contents of *A.tamarense.* All error bars indicate SE of the three replicates.*represents a statistically significant difference of *p*<0.05 when compared to the control, **represents a statistically significant difference of *p*<0.01.

To investigate whether exposure to different supernatant concentrations of BS01 affected the molecular function of algal cells, the content of total proteins, activities of antioxidant enzymes, and membrane lipid peroxidation were measured. The cellular protein contents decreased with increased treatment concentrations of supernatant (Fig. 2A). At 2 h exposure, the content of protein increased significantly (*p*<0.01) at the concentration of 0.5% and decreased with increasing concentration of BS01 supernatant. At 4 h and 8 h exposure, cellular protein contents decreased significantly (*p*<0.01) compared with the control. However, protein content showed a slightly increase compared with other groups at 12 h exposure. Significant inhibition occurred at 8 h exposure, and the protein contents of the 8 h treatment groups were decreased by 16.2, 37.4, and 43% of the control at concentrations of 0.5, 1.0, and 1.5%, respectively.

Excessive ROS may cause irreversible oxidative damage to proteins, lipids and nucleic acids, and activate signaling pathways ultimately leading to cell death [42]. ROS and lipid peroxidation levels can reflect the oxidative damage of the cellular components. Fig. 2B showed the fluorescence intensity of ROS after 2 h treatment with supernatant. Compared with the control, the ROS level was significantly (*p*<0.01) increased in response to the concentration of supernatant. Intracellular ROS levels after 2 h treatment with concentrations of 0.5, 1.0, and 1.5% supernatant were 2, 1.8 and 2.5 times those of the control, respectively. At the highest treatment concentration, ROS level was also higher than other groups, and this indicated that excessive ROS was produced in the damaged cells by the BS01 supernatant.

### Effect of Lipid Peroxidation and Antioxidative Enzyme Activities

**Figure 3:**
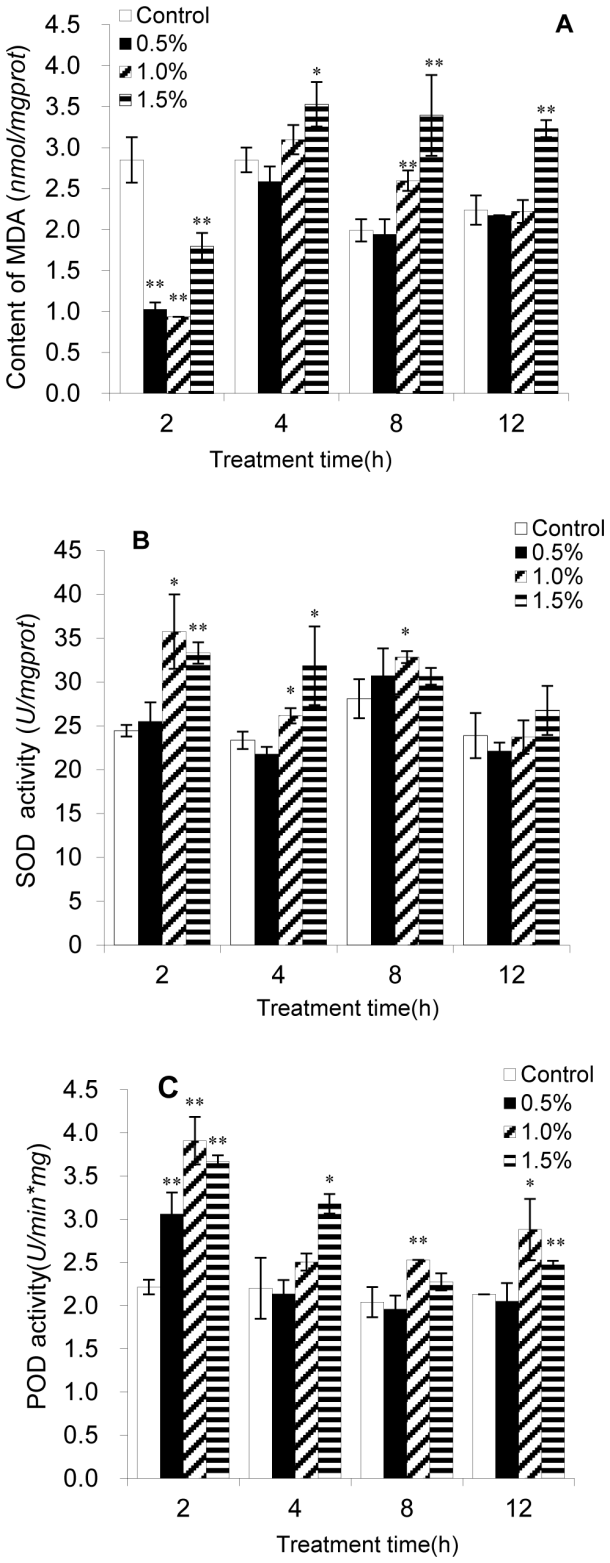
Effects of BS01 supernatant on (A) MDA, (B) SOD and (C) POD contents of *A. tamarense*. All error bars indicate SE of the three replicates.*represents a statistically significant difference of *p*<0.05 when compared to the control, **represents a statistically significant difference of *p*<0.01.

MDA is a natural biomarker produced in the reaction of lipid peroxidation (LPO) [43], which can be quantified and used for evaluation of this process. Fig. 3A showed the content of MDA. Compared with the control and other treatment groups, MDA contents in algal cells treated for 2 h were the lowest and were significantly (*p*<0.01) decreased at each concentration. As the treatment time was prolonged, the MDA levels increased and were higher than the control group at each concentration. After 4 h exposure, the MDA contents of algal cells treated with the lowest supernatant concentration (0.5%) had almost no change compared with the control, while that of the highest concentration treatment (1.5%) were all significantly (*p*<0.01) increased compared with the control. MDA levels after treatment with the 1.5% concentration for 2, 4, 8 and 12 h were 0.62, 1.24, 1.70 and 1.44 times those of the control, respectively.

Cellular enzymatic activities including SOD and POD were determined to investigate the cellular defense response induced by BS01 supernatant (Fig. 3B, C). Fig. 3B showed that the activities of SOD increased significantly compared with the control after algal cells were treated for 2 h. The activity values were 1.04, 1.46 (*p*<0.05) and 1.36 times (*p*<0.01) those of the control when algal cells were treated with 0.5, 1.0 and 1.5% of BS01 supernatant. However, SOD activities decreased after the 4 h treatment, and SOD activities recovered to the control level at 12 h exposure. In the 12 h treatment group, the value of SOD was slight higher than the control only at the 1.5% concentration, showing that longer exposure times could not induce a significant increase in the SOD activity.

POD activity showed a similar pattern to SOD activity, where activity increased after 2 h exposure, and decreased after 4 h exposure to the BS01 supernatant (Fig. 3C). The maximum POD activity was 1.77 times (*p*<0.01) that of the control, which was observed after 2 h of exposure to the 1.0% BS01 supernatant. The activity values were 1.38 (*p*<0.01), 1.77 (*p*<0.01) and 1.66 times (*p*<0.01) those of the control when algal cells were treated with 0.5, 1.0 and 1.5% of BS01 supernatant. POD showed a slightly difference in activity pattern between 8 and 12 h exposure. At 12 h exposure, although POD activity increased significantly (*p*<0.01) compared with the control at 1.5% concentration, the value was lower than the 2 h treatment group at the same treatment concentration.

### Effect of BS01 Supernatant on Pigment Contents and the Fv/Fm Ratio

**Figure 4:**
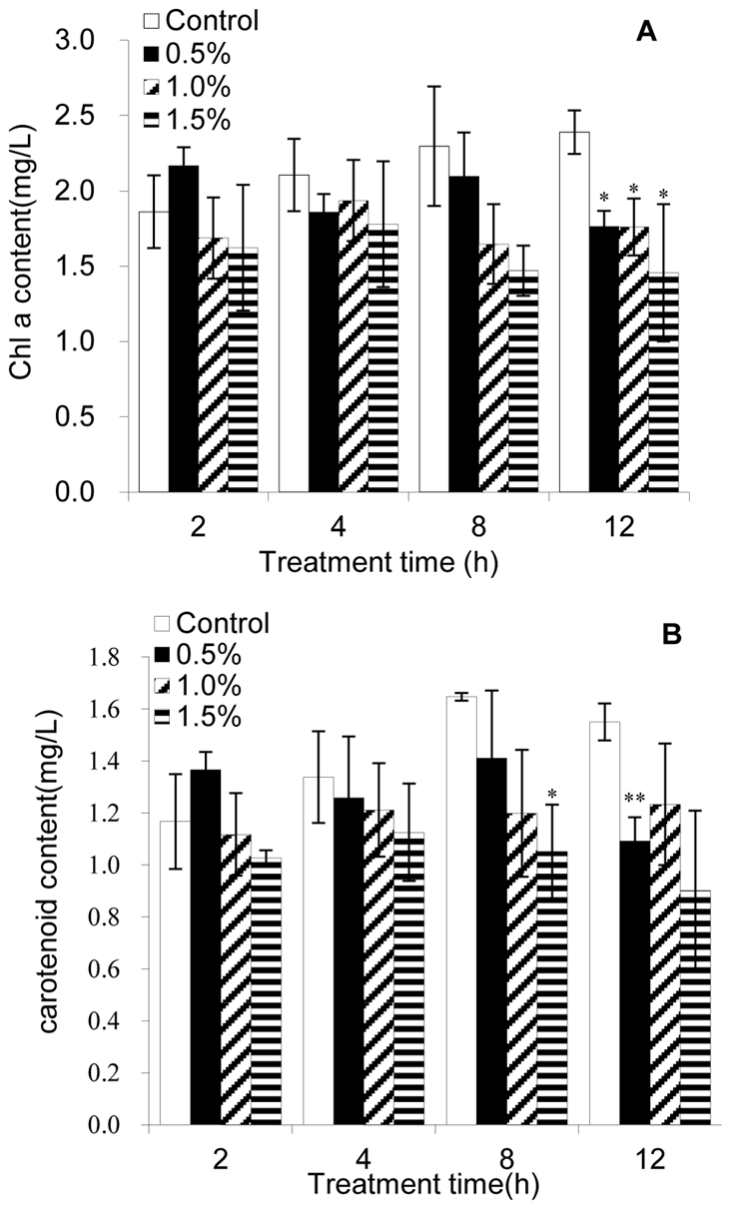
Inhibitory effects of BS01 supernatant on chlorophyll a and carotenoid content in *A.tamarense*. All error bars indicate SE of the three replicates.*represents a statistically significant difference of *p*<0.05 when compared to the control, **represents a statistically significant difference of *p*<0.01.

The inhibitory effects of BS01 supernatant on Chl a and carotenoids in *A. tamarense* increased after 2–12 h exposure (Fig. 4A, B). Compared with the control, the contents of Chl a showed no significant change in the treatment groups before 8 h. At the 8 h treatment, the contents of Chl a were slight lower than the control. At 12 h exposure, inhibitory effects were highest, and the contents of Chl a were approximately 73.8, 73.6 and 60.9% of the control after exposure to the 0.5, 1.0 and 1.5% concentrations of supernatant. When the supernatant concentration reached 1.5%, Chl a contents were lower than the other supernatant concentration treatments in each group. Fig. 4B indicated that the content of carotenoids in algal cells showed almost no change versus the control after either 2 h or 4 h exposure. After 8 h exposure to the BS01 supernatant, the carotenoid content was significantly (*p*<0.05) decreased when the supernatant concentration reached 1.5%. The carotenoid contents in the algal cells exposed to the 0.5, 1.0 and 1.5% concentrations were 85.7, 72.8 and 63.8% those of the control. At 12 h exposure, the contents of Chl a were significantly (*p*<0.05) decreased when the supernatant concentration was 0.5% and the results showed that carotenoid contents were 70.4, 79.6 and 58.2% of the control.

**Figure 5:**
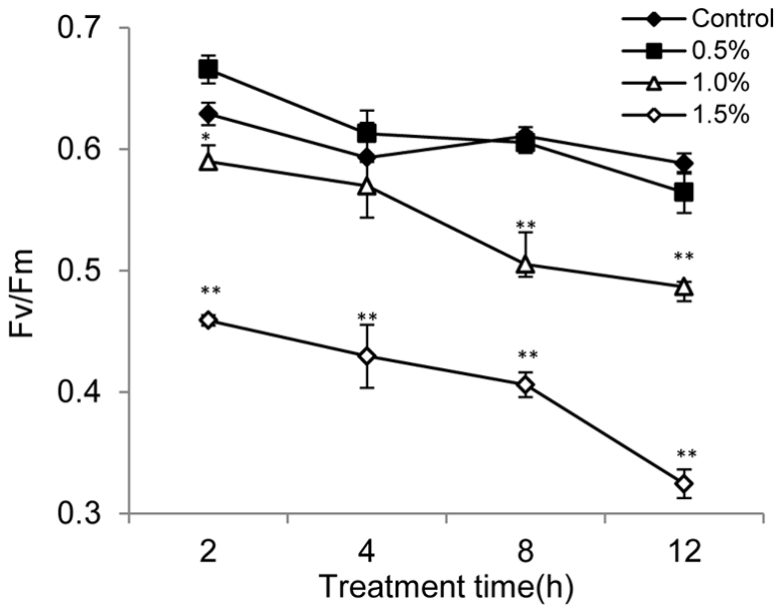
Photosynthetic efficiency (Fv/Fm) of *A.tamarense* cells treated with various concentrations of BS01 supernatant. All error bars indicate SE of the three replicates. *represents a statistically significant difference of *p*<0.05 when compared to the control, **represents a statistically significant difference of *p*<0.01.

To evaluate the photosynthetic status of the cells under the stress of BS01 supernatant, we also measured the value of the maximum photochemical quantum yield Fv/Fm. Within the 12 h treatment, the Fv/Fm ratio of the 0.5% treated cells had the same level as those in control cells (Fig. 5). We observed a lowered photosynthetic yield (Fv/Fm) in the 1.0% concentration supernatant treatment group. In the 1.5% treatment group, the Fv/Fm values decreased significantly compared with the control and this implied that inhibition of the photosynthetic yield (Fv/Fm) was caused by the supernatant.

### Effect of BS01 Supernatant on the Transcription of Photosynthesis-related Genes

**Figure 6:**
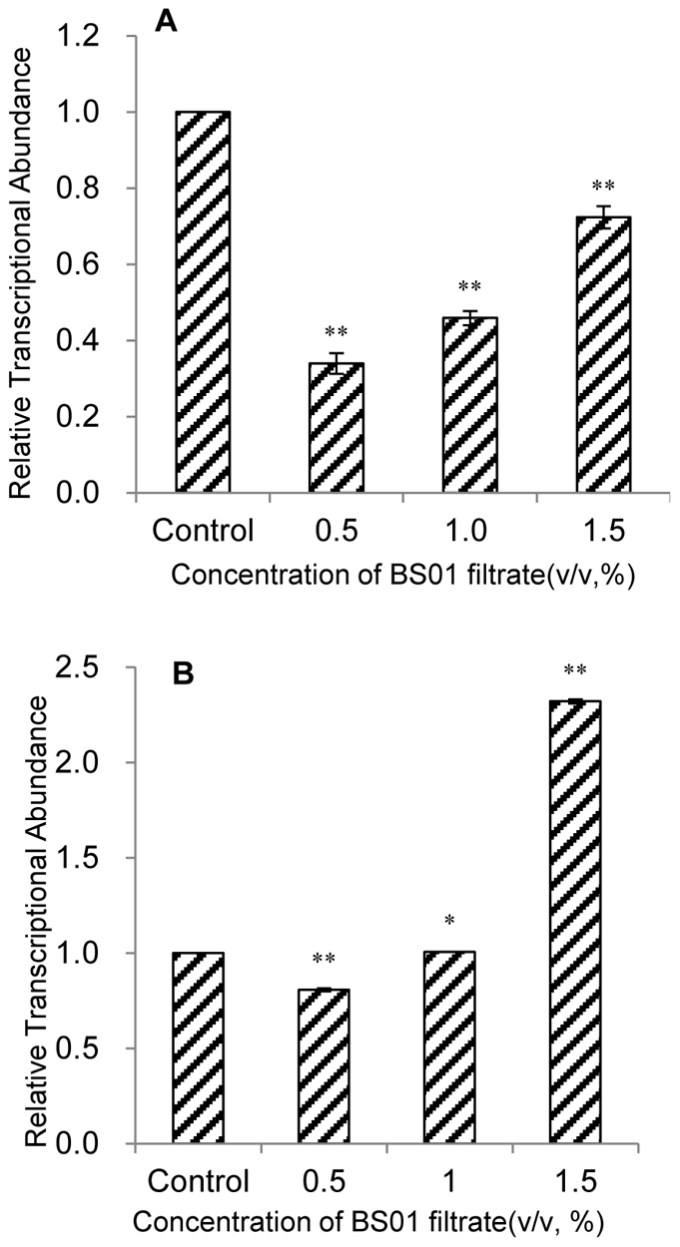
Expression of *psb*A (A) and *psb*D (B) genes in *A.tamarense* exposed to different concentrations of BS01 supernatant for 2 h. Values were normalized to levels of 18S rRNA, a reference gene, and represent the mean mRNA expression value±S.E.M. (n = 3) relative to the control. *represents a statistically significant difference of *p*<0.05 when compared to the control, **represents a statistically significant difference of *p*<0.01.

Fig. 6 showed the effects of various concentrations of BS01 supernatant on the relative transcript abundances of photosynthesis related genes after 2 h exposure. Abundance of the *psb*A transcript was significantly affected by BS01 supernatant (Fig. 6A). The expression of *psb*A showed a significant variation (*p*<0.01) at all concentrations of BS01 supernatant, but at lower concentrations it had a higher down-regulation value than at higher concentrations. The relative expressions of *psb*A were 0.34, 0.45 and 0.72 times those of the control at the 0.5, 1.0 and 1.5% concentrations. *psb*D exhibited a somewhat different response to BS01 supernatant compared with *psb*A (Fig. 6B). At the 0.5% concentration, the abundance of the *psb*D transcript decreased, and the values were 0.81 times that of the control. However, the *psb*D transcript increased at higher treatment concentrations especially at the 1.5% concentration, and the relative expression of *psb*D was 2.32 times that of the control.

## Discussion

Blooms of species of *A. tamarense* occur around world and are responsible for most incidents of PSP [44]. It is necessary to seek effective methods to control the blooms and explore the mechanism of alga lysis. There are few reports in the literature on the effects of bacteria on *A. tamarense*, and most of them only describe the relationship between alga and bacteria. To further understand the mechanism action of the BS01 supernatant on the alga, the cellular-soluble protein, ROS, lipid peroxidation products, pigment contents, Fv/Fm ratio, enzymatic antioxidants, ultrastructure changes and gene expression were determined.

In our previous study [33], we demonstrated that BS01 can significantly inhibit the growth of *A. tamarense*. Cellular-soluble protein is one of the basic indicators to reflect the physiological state of cells. The increase of cellular protein contents may imply that new protein is synthesized to resist external stress. After 2 h treatment, soluble protein content was significantly increased when 0.5% of the BS01 supernatant was added into the algal culture, but it decreased quickly when the concentration of BS01 supernatant and the treatment time increased, which implied that some of algal cells gradually lysed (Fig. 2A).

ROS including superoxide anion radicals, hydrogen peroxide and hydroxyl radicals have been historically associated with cell death [29], [45]. In most eukaryotes, the primary sources of ROS are the mitochondrial electron transport chain and the peroxisomes. However, the plant cell contains the chloroplast with an intense electron flow that leads to high rates of ROS production. Superoxide anions (O_2_•^-^) are generated as byproducts of photosynthetic electron transport and readily converted into hydrogen peroxide (H_2_O_2_) inside the chloroplast through chemical and enzymatic reactions. Singlet oxygen (^1^O_2_) and hydroxyl radicals (•OH) are also produced during photosynthesis and can cause oxidative damage [46]. The indirect damage by ROS includes lipid peroxidation, inhibition of photosynthesis, and the oxidation of photosynthetic pigments such as chlorophylls and phycobilins [47]. Our present studies showed that BS01 supernatant could significantly increase ROS level in algal cells after 2 h exposure to different concentrations of BS01 supernatant (Fig. 2B). Under lower concentrations (0.5 and 1.0%), ROS levels at 2 h were significantly (*p*<0.01) higher than that of control, which indicated that BS01 supernatant increased intracellular ROS significantly in a short time. With increasing concentration of the supernatant, ROS level was much higher than in the treatment group with lower concentration. This phenomenon implied that BS01 supernatant in high concentration induced algal cells to produce redundant ROS, and this might not be totally cleared by the algal cells and might eventually cause cell damage. As ROS increased dramatically in a short time, lipid peroxidation in the cells increased after 4 h, which further strengthened the extent of cellular damage.

MDA is an indicator of the lipid peroxidation which reflects cellular oxidative damage. Cell membranes are made of unsaturated phospholipids and are vulnerable to oxygen radical attack. Through the direct assay of the MDA content, we confirmed that there was a significant elevation in MDA content accompanying BS01 supernatant concentration and duration of exposure. As seen in Fig. 3A, the MDA level in the algae after 2 h exposure was significantly decreased (*p*<0.01), and it is possible that the cell cleared MDA quickly by metabolism. However, with ROS accumulation and time passing, the MDA level was increased after 4 h. At the 1.5% concentration, the MDA level was higher than that at the lower concentrations, and this implied that algal cells experienced more serious oxidative damage. This test demonstrated the consequences of increasing ROS and confirmed the oxidative damage on the cellular membrane. To handle environmental stresses, algal cells can activate various oxidases and PODs by intracellular ROS. Organisms possess ROS scavengers, such as SOD, CAT and POD, which protect against the potential damaging effects of ROS [38], [48]. Our results indicated that algal cellular antioxidant enzymes were triggered to different degrees when cells were exposed to BS01 supernatant. SOD activities were enhanced significantly in 2 h when exposed to the 1.0% concentration or above, but only had a slight change in 12 h. This showed that SOD activities were activated in a short time. However, SOD activities were returned to the normal level after a longer time exposure of 12 h, which implied that the BS01 stress in algal cells was relieved (Fig. 3B). Similarly, the increased activities of POD indicated potential protection against oxidation by these antioxidant enzymes. Under normal environmental conditions, the production and scavenging by ROS are in dynamic homeostasis and the excess ROS cannot be accumulated in the algal cells, and so the cells grow and develop normally. However, when the cells suffer from external stress such as drought, salinity, high light intensity and UV [49]–[52], ROS metabolism in severe imbalance, resulting in excessive ROS accumulation in the cells, could cause severe damage to the cells. Enzymic antioxidant systems are the most important defense systems to protect cells to avoid environmental stress. PCB induces the increase of SOD activity in dinoflagellates [30] and the increase of POD in green alga under the stress of glufosinate [43]. In our present work, enzymes of SOD and POD were seen to be directly involed in resisting BS01 stress in algae.

Photosynthesis depends on the absorption of sun light by chlorophyll molecules in PSI and PSII. Chl a is one of the primary light-harvesting chromoproteins in algae and has an important role in algal photosynthesis. Plants and algae are able to synthesize specific pigments in the chloroplast called carotenoids to prevent photo-oxidative damage caused by the highly reactive by-products of photosynthesis [53]. During our experiments, Chl a content was lower than that of the control even at low concentration of the supernatant, especially at 12 h exposure (Fig. 4A). This indicated that the ability of the cells to synthesize chlorophyll was decreased [54]. Moreover, the lower chlorophyll content indicated a decrease in the antenna size of the photosynthetic reaction centre complexes [55]. The results for carotenoid content had a similar trend compared to the Chl a content. At 12 h exposure, the carotenoid content was significantly decreased (*p*<0.01) compared with the control, and it might imply that the chloroplast was seriously damaged so that it could not synthesize enough carotenoids to resist oxidative damage. Another indicator of the ability of photosynthesis Fv/Fm was detected (Fig. 5). The Fv/Fm value decreased as the concentration of BS01 supernatant increased. This result indicated that the PSII system could not have a normal photochemical reaction under external stress.

Although little ultrastructural information of dinoflagellates has been reported, we could also detect ultrastructural changes induced by BS01 compared with normal cells (Fig. 1). It could be seen that cells experienced extreme plasmolysis and vacuolization under the stress of BS01, but the mitochondria and vacuoles were not affected in these cells. However, the chloroplasts were not similar to these organelles. Chloroplasts contain a highly organized thylakoid membrane system and provide all structural properties for optimal light harvesting [56]. The damage of chloroplasts can disrupt normal photosynthesis and induce algal death. TEM analysis showed that BS01 supernatant partially damaged the structure of the chloroplasts, which might also induce the decrease of pigments. It is likely that the structural damage to the chloroplasts induced by BS01 supernatant was accompanied by a reduction in photosynthetic activity and gene expression.

To explore whether there was damage to the photosynthetic processes, the *psb*A and *psb*D genes were selected as target genes in this research. *psb*A and *psb*D encode two core proteins, D1 and D2 of PSII, respectively [57]. As is true for the other chloroplast-encoded PSII proteins, both Dl and D2 are highly conserved in all species whose genes for these proteins have been sequenced. The decrease in photosynthesis related gene transcription could block electron transport and result in generating of ROS. The abundance of the *psb*A gene was significantly decreased in response to BS01 supernatant (Fig. 6). However, as the concentration of the BS01 supernatant increased, *psb*A gene expression was increased but it was also lower than that in the control. However, the *psb*D gene was up-regulated at the 1.0 and 1.5% concentrations compared with the control. This suggested that the degree of repair for PSII was increased as the concentration of BS01 supernatant increased. Although the abundance of the *psb*D was increased at high concentration, Fv/Fm values were still decreased. The increased expression of *psb*D might be associated with the repair of PSII. We could not observe photoinhibition if the repair rate was equal to the photodamage rate [32], [58]. Our studies indicated that the repair rate did not keep up with the damage rate under the stress of BS01 supernatant. We also obtained another result which was that the *psb*A and *psb*D genes had different responses to BS01 supernatant, and the mechanism of this is still not clear.

### Conclusions

The results from our present work suggested that *Brevibacterium* sp. BS01 supernatant altered physiological state, enzymic antioxidant systems, pigment content, subcellular structure and gene expression of photosynthetic in *A. tamarense*. To our knowledge, there is little information concerning the effects of an algicidal bacterium on an aquatic organism such as *A. tamarense*. Our studies demonstrated that BS01 supernatant inhibited photosynthesis related gene transcription, which might have blocked the electron transport chain of PSII to form ROS. The increase of ROS level destroyed the membrane and pigment synthesis, activated enzymic antioxidant systems, and altered the ultrastructure of the cells, ultimately inducing cell death.
